# Language Assistance Services in Nonfederally Funded Safety-Net Medical Clinics in the United States

**DOI:** 10.1089/heq.2021.0103

**Published:** 2022-01-20

**Authors:** Vicki L. Denson, Janessa M. Graves

**Affiliations:** ^1^ College of Nursing, Washington State University, Vancouver, Washington, USA.; ^2^ College of Nursing, Washington State University, Spokane, Washington, USA.

**Keywords:** limited English proficiency, interpreters, safety-net clinics

## Abstract

**Background:** In the United States, nonfederally funded safety-net clinics provide health care services to underserved populations, including patients with limited English proficiency. Unlike clinics that receive federal funding, which requires provision of qualified interpreters, these clinics are not required to provide such services.

**Objective:** The aim of this study was to describe the types of language assistance services used by safety-net clinics and their approaches to medical interpreter training for volunteers and staff.

**Methods:** A survey was administered by mail and email to nonfederally funded medical safety-net clinics identified from publicly available directories. The survey collected information on clinic characteristics, interpreter modalities used, and interpreter training and could be completed on paper or online.

**Results:** Among 859 eligible clinics, 216 completed the survey (24% response rate). Few clinics reported timely access to professional interpreter services in-person (18.5%), by phone (23%), or by video (7%), while 80% of clinics used *ad hoc* family member or friend to interpret and 53% used *ad hoc* child to interpreter. Seventy-eight percent of clinics reported using bilingual staff, providers, and/or volunteers. Staff/volunteer training was provided by 22 clinics (11%).

**Conclusion:** Most safety-net clinics relied upon *ad hoc* interpreters, contrary to best practices. Use of *ad hoc* interpreters can lead to errors in interpretation, contributing to inequities in quality of health care services. Future efforts should identify economical strategies to improve access to qualified interpreter services at nonfederally funded safety-net clinics.

## Introduction

Language diversity has rapidly changed over the last three decades in the United States, with ∼67.2 million individuals aged 5 years and older speaking a language other than English at home.^1^ Of those individuals, 38% are classified as having limited English proficiency (LEP) or speaking English less than “very well.”

Several modalities exist to communicate with patients with LEP, including (1) the use of trained medical interpreters, (2) direct communication with bilingual health care providers and/or staff, (3) use of *ad hoc* interpreters, and (4) use of digital translation applications. *Ad hoc* interpreters, or lay interpreters, are untrained individuals who are called upon to interpret and may include a patient's family member or child, friend, bilingual staff member who is asked to interpret outside of their assigned duties, or a self-declared bilingual individual who volunteers to interpret.^2^

However, *ad hoc* interpreter's inaccuracies have been documented as answering for the patient, substituting, adding, or omitting information during interpretations,^3^ which can increase the risk for individuals with LEP to experience adverse events such as increased risk of error with verbal consent process,^4^ longer hospital stays, including surgical infections, falls, and pressure ulcers, as well as greater chance of hospital readmissions for chronic conditions^5^; whereas language concordant care has been found to improve patient outcomes such as better glycemic control of diabetes, control of hypertension, and adherence to treatment and screening procedures.^6^ Patient's rights include receiving information that he or she can understand and is critical for an informed consent process to ensure safety.^5^

Federal mandates, such as Title VI of the 1964 Civil Rights Act and Executive Order 13166, Improving Access to Services for Persons with LEP, have been issued to ensure equitable access to health care services in the US, irrespective of language assistance needs.^7^ These initiatives provide specific protections for individuals with LEP and promote effective, culturally competent communication between providers and patients with LEP. Under these policies, federally funded clinics must provide meaningful language assistance services for individuals with LEP at no cost, as well as ensure that their interpreters are competent to provide language assistance.^7,8^

To provide high-quality language assistance services, many organizations rely upon professional interpreter services, which can be costly. The fees associated with professional interpreter services vary between the mode of service delivery, ranging from $45 to 150 per hour for in-person interpretation, $1.25–3.00 per minute for telephonic interpretation, and $1.95–3.49 per minute for video remote interpreting with additional costs for setup and/or minimum time requirements.^9^ Interpreter service costs are sometimes reimbursed by private insurance or federally funded health insurance programs, such as Medicaid.^9^

For clinics that do not receive or rely upon federal funding, including free, charitable, and faith-based clinics, reimbursement for professional interpreter services is often not feasible, as their patients are uninsured or underinsured and receive services at no cost or for reduced fees, such as a sliding fee scale or donation. The high cost of professional interpreter services may render their use within safety-net clinics economically impractical.^10^

The inability for many free and charitable clinics to provide professional interpreter services presents a concerning problem, as these clinics provide critical support as part of America's safety-net health care system. In 2019, free and charitable clinics and pharmacies served an estimated 782,000 new patients and 2 million unduplicated patients across 2,000 communities, the majority of which were in medically underserved areas.^11^ These safety-net clinics save U.S. emergency departments ∼$9.6 billion dollars annually.

Unlike Federally Qualified Health Centers (FQHCs) that are staffed by paid clinicians, free and charitable clinics rely mainly on private funding and a volunteer/staff model.^12^ This approach includes utilizing volunteer or *ad hoc* interpreters to bridge the gap in communication between providers and patients with LEP.

Previous studies have focused on provision of linguistically appropriate care in hospitals, emergency department settings, and federally funded clinics; however, scant studies have specifically examined interpreter services offered in nonfederally funded free and charitable clinics. To our knowledge, no studies have explored faith-based free and charitable clinics where provision of services is often hindered due to limited financial resources and low access to qualified, professional medical interpreters. The purpose of this study was to describe the types of language assistance services used by nonfederally funded free, charitable, and faith-based medical clinics in the United States and explore their approaches to medical interpreter training.

## Methods

### Study design and sample

This cross-sectional study was conducted from October 2019 to March 2020 and involved a survey of nonfederally funded free, charitable, and faith-based medical clinics in the United States.

The study sample was derived from clinics listed in the publicly available directories of The National Association of Free & Charitable Clinics and the Christian Community Health Fellowship. Inclusion criteria included indication of providing free and/or charitable primary medical care services and location within the United States or District of Columbia. Title X family planning clinics and FQHCs were excluded from the study as receipt of federal funding requires provision of meaningful language assistance services at no cost. Clinic names, addresses, and phone numbers were obtained from the directories. The name and email address for the clinic medical director or manager were obtained from clinic websites, if available.

### Survey instrument and measures

Information on clinic operations were collected using an 11-item survey with multiple choice, fill-in-the-blank, and open-ended questions related to clinic characteristics, interpreter services modalities, and training of interpreters.

Clinic characteristics included the estimated percent population with LEP seen within the last year and type of health services provided (i.e., medical, dental). Clinics reported their funding sources, which could include private sector donations, nonfederal grants, or provision of services for free or for a minimal fee (only if fee(s) waived for essential services). Interpreter service modalities included child of patient, family or friend of patient, bilingual staff, provider, volunteer, professional video remote interpreting, professional interpreting via phone, and/or professional interpreting in person.

Clinics were asked to list any digital communication tools (e.g., mobile applications or websites) used in the clinic via an open-ended question. Clinics indicated if they provided medical interpreter training for staff, providers, and/or volunteers; respondents also described the type of language assistance training offered in an open-ended question. Survey items were pilot-tested and iteratively revised in partnership with a local clinic manager before distribution to ensure appropriateness and usability.

### Survey administration

The paper survey, with an introduction letter describing the study, return address, and postage were mailed to clinic managers or medical directors for all clinics that met inclusion criteria. Clinics with an identified email address also received an email with an introduction letter and link to a Qualtrics online survey. All clinics had the option of completing the survey online or by mail on paper (the letter mailed to the clinics included a link to the online survey); clinics were asked to complete only one survey (either mode).

Consent was assumed with initiation of the survey. One follow-up postcard and one follow-up email (for valid email addresses) were sent to all clinics that did not respond after 4 weeks. The initial mailing (or email) and reminder were distributed October 9 and November 4, 2019. Data collection ended on March 1, 2020. No personal identifiers of respondents were collected. Clinic names were collected to monitor responses, then separated from the data. As a token of appreciation for participation, clinics could choose to enter a voluntary raffle for one of six $50 Amazon.com e-gift cards.

### Analysis

We used IBM SPSS Statistics (version 24) for all analyses. Data from Qualtrics survey database were exported to SPSS, while data from paper surveys were manually entered into SPSS by the primary investigator. Analyses involved descriptive statistics, including frequencies and percentages to describe the characteristics of the clinics, languages spoken by patients and clinic staff/volunteers/providers, language assistance services used, digital tools used for communication, and provision of medical interpreter training. Pearson chi-square and/or Fisher's exact tests were used to compare the proportion of clinic patients with LEP and language assistance services used.

Clinics' description of types of medical interpreter training provided was categorized based on whether training was done in-house, in partnership with an outside agency, or a combination of both. The Washington State University Office of Research Assurances has found that the project is exempt from the need for human subject review.

## Results

A total of 922 clinics met the study inclusion criteria and were sent surveys by mail. A subset of these clinics with email addresses (*n*=207) were also sent an email with a link to the web-based survey. Sixty-seven mailed surveys were returned as undeliverable or vacant. Of these undeliverable surveys, four had a valid email address resulting in a valid sample of 859 clinics. A total of 73 clinics responded by via Qualtrics online survey and 171 responded by mail. Three clinics completed both online and paper versions of the survey; the responses dated earlier (per online timestamp or postmark) were retained.

Twenty-five surveys were deemed ineligible for the following reasons: identified as a FQHC (*n*=3), provided only dental services (*n*=1), served zero patients with LEP (*n*=7), and reported that they received funding by federal government grant, Medicare, and Medicaid (*n*=14). After excluding duplicates and responses from ineligible clinics, a total of 216 returned surveys were deemed valid (24% response rate).

### Clinic characteristics

Surveys were obtained from clinics across from 42 states and the District of Columbia. All clinics provided medical care; 71 (33.3%) also reported providing dental care. The majority (*n*=150, 69.4%) reported private sector funding (e. g. donations, nonfederal grants) and 118 (55.6%) reported providing services for free or for minimal fee(s) with fee(s) waived for essential services (categories were not mutually exclusive). Ninety-two (43.1%) of respondent clinics were identified as faith based. All clinics reported seeing patients with LEP during the last year (Table 1).

**Table 1.** Percentage of Patients with Limited English Proficiency Seen Within Last Year

Patients with LEP (%)	Clinics *n* (%)
1–25	100 (46.3)
26–50	64 (29.6)
51–75	29 (13.5)
76–100	23 (10.6)

LEP, limited English proficiency.

### Languages spoken

**FIG. 1.**
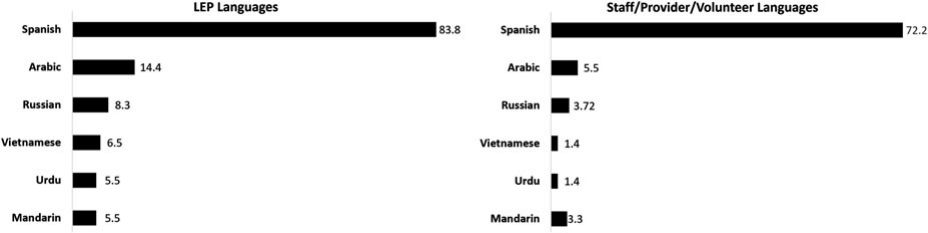
Percent of nonfederally funded safety-net clinics reporting the most common language spoken by their patients with LEP and among staff, providers, and volunteer, 2020 (*N*=216). Values indicate percent of clinics; language categories are not mutually exclusive. LEP, limited English proficiency.

Clinics reported that most non-English-speaking patients spoke Spanish (*n*=181, 83.8%). Arabic and Russian were also commonly reported as being spoken by patients at 14.4% and 8.3% of clinics, respectively (Fig. 1).

One hundred seventy-eight clinics (82.4%) reported their staff, providers, and/or volunteers spoke a language other than English. Most clinics reported Spanish as the spoken language other than English by staff, providers, and/or volunteers (*n*=156, 72.2%) (Fig. 1). Nearly all clinics with large patient volume with LEP reported having staff, providers, and/or volunteers who spoke a language other than English, which was significantly more than clinics with lower LEP patient volume (Table 2).

**Table 2.** Language Assistance Services in Clinics with High and Low Proportions of Patients with Limited English Proficiency Seen Within Last Year

	Clinic population of patients with LEP		Significance (*p*-value)
	0–50% *n* (%)	>50% *n* (%)	
Do the staff, providers, and/or volunteers at your clinic speak any language other than English?			0.003^*^
Yes	129 (78.7)	49 (96.1)	
No	35 (21.3)	2 (3.9)	
Does your clinic provide medical interpreter training for staff, providers, and/or volunteers?			0.001^**^
Yes	10 (6.4)	12 (25.5)	
No	146 (93.6)	35 (74.5)	

Percentages indicate column percent.

^*^ Fisher's exact test.

^**^ Pearson chi-square test.

One hundred sixty-one clinics (74.5%) responded to the question asking to rate the fluency of staff, providers, and/or volunteers. Among these, 67.1% (*n*=108) reported that their staff, providers, and/or volunteers had non-English language fluency ranging from “near-native fluency” to “native speaker,” 20.5% (*n*=33) reported “conversational” fluency, and 12.4% (*n*=20) reported “beginner” fluency among their staff, providers, and/or volunteers.

### Summary of language assistance services used

**FIG. 2.**
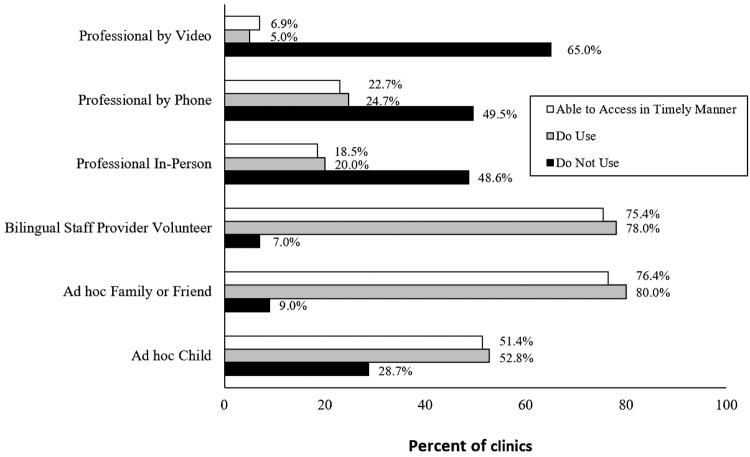
Language assistance services used by nonfederally funded safety-net clinics in the United States, 2020 (*N*=216).

The most used language assistance services offered by clinics were (1) bilingual staff, providers, or volunteers; (2) interpretation conducted *ad hoc* by an adult family member or friend, or (3) interpretation conducted *ad hoc* by a child (Fig. 2).

Access to language assistance services in a timely manner for LEP patients included (1) use of a patient's child; (2) family member or friend of a patient; (3) bilingual staff, provider, or volunteer; or (4) professional video remote interpreting, professional interpreting via phone, and/or professional interpreting in person. One hundred eleven (51.4%) clinics indicated that they were able to access a child of the patient as an interpreter in a timely manner, while 165 (76.4%) were able to access a family or friend in a timely manner. One hundred sixty-three (75.4%) clinics were able to access bilingual staff, providers, or volunteers in a timely manner.

Only 15 clinics (6.9%) reported that they were able to access professional video remote interpreting, 40 clinics (18.5%) were able to access professional interpreting in person, and 49 clinics (22.7%) were able to access professional interpreting via phone in a timely manner (Fig. 2).

### Digital tools used for communication

Among the 204 clinics that responded to the question about use of digital tools (e.g., mobile app or website) to communicate with patients with LEP, 121 (59.6%) reported use of these tools, while 82 (40.4%) of clinics did not use digital tools. The most common digital tool reportedly used was Google Translate (*n*=103, 48%). Other types of digital tools were reported by three or fewer clinics (e.g., iTranslate Converse, Language Line, Microsoft Translator).

### Provision of medical interpreter training

Among the clinics that reported information about medical interpreter training (*n*=203), 22 (11%) indicated that the clinic provides some type of medical interpreter training for staff, providers, and/or volunteers. Nearly four times as many clinics with over half of patients with LEP reported offering interpreter training compared to clinics with less than half patients with LEP (Table 2).

Types of training included in-house training created by the clinic (*n*=15), in-house training created by an outside organization (*n*=4), and a combination of in-house and outside organization training (*n*=4). Four clinics reported that training included passing an examination or written test to determine language competency and skill level, with one clinic's testing leading to certification as an interpreter.

Alternative approaches of training were also used by clinics. For example, two that did not provide training through the clinic reported reimbursing volunteers or staff who obtained certification as medical interpreters at outside agencies. Another clinic reported reimbursing a nurse for taking a community college Spanish course. Another stated that a local organization certified the clinic's staff/volunteers as a certified medical interpreter for half the cost if the staff/volunteer committed to serving at the clinic.

## Discussion

Effective communication between patients with LEP and health care providers is essential for provision of quality care. This national survey focused specifically on language assistance services and interpreter training within nonfederally funded free, charitable, and faith-based clinics.

Clinics reported the types of language assistance services used to communicate with patients with LEP. Our findings suggest that most clinics rely on the use of *ad hoc* interpreters and few clinics utilize professional interpreter services, consistent with previous research based on provider and institutional self-reported practices and patient-reported experiences.^13–15^ Patients have reported satisfaction with *ad hoc* interpreters who spoke the same language, had knowledge of U.S. culture, and who had the ability to communicate their needs.^16^ However, the use of *ad hoc* interpreters often results in errors of clinical consequence compared to professional medical interpreter use.^3,17–19^

Without access to professional interpreter services, some providers rely on digital language assistance services to communicate with patients with LEP.^20^ Most clinics in our study reported use of Google Translate, a highly rated application for communicating with patients with LEP.^21^ Although the use of this digital app may be economical and convenient when in-person, phone, or video interpretation is not available, there are concerns related to the accuracy of translation leading to safety risks.

Studies have shown that with sentence complexity and higher levels of reading ability, Google Translate made more errors in translation compared to professional medical translators.^22^ The app performed more accurately when translating from English to Spanish than translation from English to Chinese. Similarly, researchers have reported only 57.5% accuracy for medical phrase translations for Google Translate,^23^ as well as translation inaccuracies, such as better performance with Spanish than Chinese or lesser used languages.^24^

The use of interpreters is a complex issue and access to professional interpreter services alone can be cost prohibitive. The Health and Human Services Department (HHS) Office of Civil Rights (2003) revised policy guidance on Executive Order 13166 to guide programs and providers who serve individuals with LEP in determining the extent and type of meaningful language assistance services that should be provided, giving small providers considerable flexibility in determining how to fulfill their obligations.^25^ In smaller nonprofit clinics that see fewer patients with LEP than English-speaking patients, the training of bilingual staff to act as interpreters may help to reduce costs associated with professional interpreters.^25^

Given the financial constraints facing nonfederally funded clinics, strategies to assess language proficiency and train bilingual clinic staff and/or volunteers may provide the most economical solution in these clinics to facilitate efficient health care delivery and improve patient safety and outcomes; yet, our data revealed that few clinics provided some type of interpreter training for staff, providers, or volunteers. Vandervort and Melkus (2003) reported similar findings for ambulatory clinics, demonstrating a lack of screening and training of clinic staff who provided interpretation regarding ethics and skill of interpreting.^26^

Strategies to improve access to quality interpretation in safety-net clinics could include conducting a validated oral proficiency test such as the Clinician Cultural and Linguistic Assessment for staff, providers, and volunteers to evaluate their non-English language skills and provide training for those who need to increase proficiency.^27^ Efforts to teach medical conversation language skills in non-English languages, such as Spanish, in health care professional education programs, while not an ideal solution, may hold some promise.^28^ While these programs, namely medical Spanish, have been integrated in medical school curricula,^28^ efforts to teach non-English medical language skills across the health professions are expanding and should be promoted.

### Limitations

This study has limitations such as a low response rate and the study sample was based on publicly available clinic directories; therefore, our sampling approach may have been subject to undercoverage or nonresponse bias. The survey relied upon self-report and not objective observation of clinic practices, the findings may be subject to social desirability bias. However, given the high proportion of clinics that reported using *ad hoc* interpreters and translation tools that may not be optimal best practices, we believe that most respondents provided accurate information about their approaches to serving patient with LEP.

Clinics responding to the question to rate the fluency of staff, providers, and/or volunteers could represent a heterogeneous group and future work should seek to separate out the categories of staff, providers, and volunteers as these roles can provide very different services in a clinic setting. Despite these limitations, the information gleaned in this survey provides important insight into the services offered at safety-net clinics in the United States.

## Conclusion

Our findings suggest nonfederally funded free, charitable, and faith-based clinics lack timely access to professional interpreter services and primarily rely on *ad hoc* interpreters, which can lead to errors in interpretation, contributing to inequities in quality of health care services. Few clinics reported provision of medical interpreter training. Future research and outreach efforts should focus on identifying economical strategies to improve access to qualified interpreter services for patients with LEP at nonfederally funded safety-net clinics.

## Abbreviations Used

FQHC: Federally Qualified Health Center
HHS: Health and Human Services Department
LEP: limited English proficiency
